# Agreement between a new web-based and a legacy paper-based food frequency questionnaires in French-speaking Switzerland

**DOI:** 10.1186/s12937-026-01309-7

**Published:** 2026-03-27

**Authors:** Angeline Chatelan, Elsa Chevillard, Sarah T Pannen, Elaine Hillesheim, Silvia Stringhini, Mayssam Nehme, Idris Guessous, Sabine Rohrmann, Nina Steinemann, Janice Sych, Pedro Marques-Vidal

**Affiliations:** 1 Department of Nutrition and Dietetics, Geneva School of Health Sciences, HES-SO University of Applied Sciences and Arts Western Switzerland, Rue des Caroubiers 25, Carouge-Geneva, 1227 Switzerland; 2 Division of Chronic Disease Epidemiology, Epidemiology, Biostatistics and Prevention Institute, University of Zurich, Zurich, Switzerland; 3 Swiss School of Public Health, Zurich, Switzerland; 4 Division of Primary Care Medicine, Geneva University Hospitals, Geneva, Switzerland; 5 Faculty of Medicine, University of Geneva, Geneva, Switzerland; 6 School of Population and Public Health and Edwin S.H. Leong Centre for Healthy Aging, Faculty of Medicine, University of British Columbia, Vancouver, Canada; 7 Institute of Food and Beverage Innovation, ZHAW School of Life Sciences and Facility Management, Wädenswil, Switzerland; 8 Department of Medicine, Internal Medicine, Lausanne University Hospital and University of Lausanne, Lausanne, Switzerland

**Keywords:** Food frequency questionnaire, Validation study, Diet, Dietary assessment, Digital health, Internet, Epidemiology

## Abstract

**Background:**

Updated and validated tools are crucial for assessing dietary intake. In French-speaking Switzerland, the only validated food frequency questionnaire (FFQ) was a paper-based version developed using data collected in 1990s. A new Swiss electronic FFQ (eFFQ) has recently been developed based on nationally representative dietary data from 2010s. The eFFQ has been recently validated and is intended for adoption in future studies among French-speaking adults living in Switzerland. Both FFQs differ in format (paper vs. electronic), food lists, frequency categories, portion sizes, and food composition databases. We aimed to compare dietary intake estimates from the recently developed eFFQ vs. the paper-based FFQ.

**Methods:**

We recruited 75 volunteers aged 18 to 75 years (54.7% female) who completed both FFQs in random order. Intakes of energy, 10 macronutrients, four micronutrients and 28 food groups were estimated using each FFQ and compared using multiple statistical tests.

**Results:**

Group-level estimates of energy and most nutrients showed good agreement between FFQs, with differences in median intakes < 10% for 8 of 14 nutrients, including energy, protein, carbohydrates, total sugars, total fat, and saturated fatty acids (SFAs). Largest differences were observed for alcohol (+ 119% in the eFFQ), fibre (+ 37%), polyunsaturated fatty acids (+ 27%), and cholesterol (+ 26%). Spearman’s correlation coefficients (SCCs) were ≥ 0.50 for 12 of 14 nutrients, and misclassification into opposite tertiles (> 10% of participants) was observed only for SFAs (13%). Bland-Altman analysis confirmed acceptable agreement for energy and macronutrient intakes. At the food group level, the eFFQ yielded significantly higher median intakes for 14 of 28 food groups – most notably plant-based protein-rich foods and cream, fatty sauces & other fats – and lower median intakes for four groups, including vegetables and fish. Ranking agreement was acceptable for most food groups (SCCs = 0.57–0.91) but weaker for water (0.07); vegetable oil (0.42); cream, fatty sauces & other fats (0.33); and mixed dishes & soups (0.47).

**Conclusions:**

The new eFFQ demonstrated good relative agreement with the previous paper-based FFQ, particularly for ranking individuals. These findings provide valuable information for researchers and policy makers comparing results of both FFQs over time and across populations.

**Supplementary Information:**

The online version contains supplementary material available at 10.1186/s12937-026-01309-7.

## Background

Diet is a major determinant of health and is shaped by evolving social and economic factors, such as food availability, preferences, income, and cultural trends [1]. Accurate dietary assessment tools are crucial for assessing dietary intake, and thus essential for epidemiological studies investigating the associations between diet, health and disease [2–6]. Although food records and 24-hour dietary recalls are considered gold standards in dietary assessment, self-administered food frequency questionnaires (FFQs) are often used due to (i) the relatively low cost when applied at a large scale, (ii) the lower burden on the researchers (once developed) and study participants, and (iii) the covering of usual dietary habits, useful for instance to derive adherence to defined dietary patterns [4–6].

Until recently, the only validated FFQ for the adult French-speaking population of Switzerland was a paper-based FFQ developed in 1994 for the Bus Santé study in Geneva [7, 8]. Because dietary habits of the Swiss population have evolved since the 1990s [9, 10], an updated FFQ was needed. Furthermore, paper-based versions of FFQs are becoming obsolete as studies assessing dietary intake increasingly rely on electronic questionnaires [11]. The benefits of electronic FFQs over paper-based FFQs are multiple. They notably reduce the burden of data entry and related errors introduced by the coder and data processing (e.g., linkage with food composition database), while improving data quality control (e.g., automatic flagging of missing data or implausible combinations) [4, 5, 11, 12].

Between 2020 and 2025, our team developed and validated a new electronic FFQ (eFFQ) for adults aged 18 to 75 years, who were German- or French-speaking and living in Switzerland [13, 14]. This new Swiss eFFQ could be implemented in future studies assessing dietary intake in the French-speaking part of Switzerland, replacing the paper-based FFQ. However, the eFFQ and the paper-based FFQ differ in terms of (i) format (paper vs. electronic), (ii) food lists based on dietary habits from the 1990s vs. the 2010s, (iii) suggested frequency categories, (iv) defined portion sizes, and (v) food composition databases used for linkage with food items. These variations in questionnaire design – together with expected shifts in dietary patterns, food availability, and consumer habits over more than two decades – are likely to lead to differences in dietary intakes estimated by both FFQs. While some differences are anticipated due to both methodological updates and contextual changes, examining their magnitude and direction is essential for understanding how variations in design influence intake estimates. This is particularly relevant for researchers and policy makers comparing results over time (longitudinal studies transiting to the new eFFQ) and across populations (studies using different FFQs). Our study aimed to compare the results obtained from the recently developed eFFQ with those from the old paper-based FFQ in a sample of volunteers, focusing on differences in total daily intakes of energy, nutrients, and food groups, and to discuss the potential methodological sources of these differences.

## Methods

### Study participants and design

Between January and April 2023, we recruited volunteers aged 18–75 years who had maintained stable body weight (≤ 10% change) and consistent dietary habits during the previous three months, had no major dietary restrictions (e.g., multiple food allergies affecting several food groups), were not using medications known to influence body weight, and were not pregnant. Eligible participants were French-speaking, had lived in the French-speaking region of Switzerland for at least one year, and had access to the internet. Volunteers were recruited through multiple outreach methods, such as social media announcements, flyers, posters, email invitations, and personal referrals. Eligible individuals were selectively invited to participate to achieve a predefined balanced sample by sex (50% females, 50% males) and age group (33% aged 18–37 years, 33% aged 38–57 years, and 33% aged 58–75 years).

Participants were asked to complete three questionnaires: (i) an online questionnaire to provide information about their sociodemographic and lifestyle characteristics using REDCap, (ii) the paper-based FFQ received by post, and (iii) the eFFQ using the link via email. The eFFQ could be completed using either a computer or a smartphone. For all online questionnaires, responses to all items were mandatory before submission. For the paper-based FFQ, a research assistant quickly reviewed each returned questionnaire upon receipt. In cases of missing data, participants were contacted whenever possible to obtain the missing information. We instructed participants to complete both FFQs within the same week and explicitly asked them not to compare their responses between the two instruments. To minimise potential order effect and reduce bias due to learning or fatigue [15], we randomised whether participants had to complete the eFFQ before or after the paper-based FFQ, as done elsewhere [16]. Participants who completed the paper‑based FFQ first (*n* = 34) had a median interval of 4.5 days between completions (range: 0–16 days), whereas those completing the eFFQ first (*n* = 39) had a median interval of 3.0 days (range: 0–23 days). Seven participants completed both FFQs on the same day, and two did not provide a completion date for the paper‑based FFQ. Among participants with distinct completion dates (*n* = 66), all adhered to the randomised order.

### Sample size

The primary outcome was the assessment of agreement in energy intake (EI) between the two FFQs, evaluated using the Bland-Altman method [6, 15, 17]. The required sample size was estimated at minimum 45 participants, based on a significance level of α = 0.05 (Type I error) and a statistical power of 80% (β = 0.20). This calculation assumed an expected mean difference (µ) of 150 kcal, a maximum allowable mean difference (δ) of 1500 kcal between the two FFQs, and a standard deviation (σ) of 500 kcal for the paired differences [17]. Of note, for dietary assessment validation studies, a sample size of at least 50 participants is usually required [15].

### Bus Santé paper-based FFQ

The self-administered, paper-based FFQ was developed and validated in 1994 using data from a single 24-hour dietary recall collected in 1991–1992 from a representative sample of 626 adults aged ≥ 35 years residing in Geneva, Switzerland [7], for the Bus Santé study [8]. From the 266 food items identified during the interviews, 141 were retained so that they accounted for more than 90% of total intake of energy, protein, carbohydrates, total fat, cholesterol, alcohol, retinol, vitamin D, and calcium, and more than 85% of fibre, β-carotene and iron. Items that did not significantly contribute to energy or nutrient intake but were frequently consumed, such as coffee and artificial sweeteners, were also included. The final food list was then condensed to 93 unprocessed and processed food items (excluding dietary supplements) based on similarities in nutritional composition. Validation was performed in a subsample of 56 individuals (51.8% female) randomly selected from the original cohort (*n* = 626), stratified by age, sex, and nationality [7]. The FFQ was considered an appropriate tool for estimating nutrient intake in the adult population of Canton Geneva, although it slightly underestimated absolute intakes of fibre, alcohol, and calcium. In 1995, the questionnaire was further refined to improve the frequency categories for certain foods and to define portion sizes according to sex [18].

The paper-based FFQ has a brief explanation on how to complete it and assesses food consumption within the previous four weeks. It is available in French, albeit unvalidated versions in English and German also exist, and has been used in several studies, such as the CoLaus|PsyCoLaus cohort study since 2009 [19] and the Swiss Childhood Cancer Survivor Study in 2021 [20]. The FFQ has seven consumption frequency categories: never, 1 x/month, 2–3 x/month, 1–2 x/week, 3–4 x/week, 1 x/day, and ≥ 2 x/day, which can be adapted according to the food items [18]. Portion sizes are divided into three size categories – smaller than, equal to, or larger than a reference portion – defined by gram weights or household measure equivalents, without the use of images. These reference portions correspond to sex-specific median values derived from the validation study [7, 18]. The smaller and larger portion sizes represent 0.5 and 1.5 times the median values, respectively, and were used to estimate daily food intake.

For the present study, participants’ responses for frequency and portion size were manually coded for each food item using a spreadsheet. Prior to intake estimation, the coded data underwent an additional quality‑control review to detect duplicate entries, assess missingness, and identify inconsistencies (e.g., a portion size recorded for an item marked as “never” consumed). When needed, the original paper questionnaire was consulted to resolve discrepancies. The daily amount of each food consumed (g/day) was calculated by multiplying the daily frequency (e.g., 3–4 x/week = 3.5 ÷ 7 = 0.5 x/day) by the corresponding sex‑specific portion size. Energy and nutrient (10 macronutrients and four micronutrients) intakes were then estimated by linking the calculated daily food amounts with the nutrient reference sheet compiled using data from the French [21] and Swiss [22] food composition databases available in 1990s. Intake estimation and quality‑control checks were performed in STATA version 16 (StataCorp, College Station, TX, USA).

### Swiss electronic FFQ

The self-administered Swiss eFFQ was previously developed using a data-driven approach [14], based on food and beverage consumption data from the first Swiss National Nutrition Survey, menuCH (2014–2015) [23]. The survey included a nationally representative sample of 2,085 adults (54.6% female, 53.5% normal weight) aged 18 to 75 years and assessed dietary intake through two non-consecutive 24-hour dietary recalls conducted 2 to 6 weeks apart [23]. The eFFQ was designed to semi-quantitatively assess dietary intake over the previous four weeks among individuals in the same 18 to 75-year-old age range, living in any of the three Swiss linguistic regions (German-, French-, and Italian-speaking). Currently, the tool is available and validated in French and German.

For its development, a total of 3,804 individual foods, mixed dishes, and beverage codes were manually standardized into 1,733 foods and subsequently grouped based on nutritional similarities, resulting in 166 main food items. A stepwise regression analysis was then applied to identify foods that together accounted for more than 90% of the between-person variance in the intake of energy or any of six selected nutrients (protein, carbohydrates, fibre, total fat, saturated fatty acids (SFAs), and vitamin C), either in the overall sample or within any of the three main linguistic regions of Switzerland. The cumulative contribution of the food items included in the eFFQ to the absolute intake of energy and nutrients, as derived from the menuCH data, was subsequently assessed in the overall sample and by linguistic region. The lowest coverage was observed for vitamin C in the French-speaking region, accounting for 89.8% of total intake.

The eFFQ is a web-based tool that, excluding dietary supplements, contains 83 main food items, of which 16 have sub-items (e.g., espresso, latte macchiato for the food item “coffee”), bringing the total to 113 items. The eFFQ contains 10 frequency categories: never, 1 x/month, 2-3 x/month, 1-2 x/week, 3-4 x/week, 5-6 x/week, 1 x/day, 2-3 x/day, 4-5 x/day, and >5 x/day. A single standard portion size was defined for each food item by aggregating intakes across eating occasions and subsequently calculating the median intake in the overall menuCH sample, weighted by age group, sex, major region, marital status, nationality, and household size. A picture per item illustrates standard portion sizes to participants. For each food item, the estimated daily amount consumed was calculated by multiplying the individual frequency of consumption (over four weeks) by the standard portion size and dividing by 28.

Throughout the eFFQ development, food lists and portion sizes were checked by nutritionists for plausibility, and pilot versions were pre-tested with researchers, nutrition experts, and lay individuals. At the start of the eFFQ, participants receive detailed instructions on completing the questionnaire. A preliminary question about vegetarian or vegan diets is included, which triggers the omission of certain food items. The eFFQ web platform automatically calculates total daily intakes of energy, 10 macronutrients and 28 micronutrients by linking more than 75% of the food items to the Swiss food composition database (version 6.4) [22] or, when items are unavailable, to the German database (version 3.02) [24]. The Swiss eFFQ includes several built‑in and protocol‑driven quality checks. All questions are mandatory, preventing missing frequency at submission. The web platform applies real‑time logic controls, including standardized frequency categories, fixed portion sizes based on population‑specific reference values, and automated calculations of daily intakes. Potential duplicate entries were verified by a research assistant.

In 2023, our team conducted a validation study of the eFFQ by comparing its results with those of 4-day food records among 177 adults living in Switzerland for more than one year and residing in the Zurich and Geneva areas (a larger sample than this study with same inclusion/exclusion criteria) [13]. The eFFQ performed particularly well for macronutrients, including protein, carbohydrates, fibre, total fat, monounsaturated fatty acids, and alcohol, as well as some micronutrients such as vitamin B12 and vitamin E, with group-level bias < 10%. Additionally, 31 of the 36 nutrients assessed had < 10% of participants classified in the opposite quartile. For food groups, those consumed in larger quantities and daily, such as meat, dairy, grains, and bakery products, showed higher correlations and ranking ability. More information about the development and validation of the Swiss eFFQ can be found here [13, 14].

### Sociodemographic and lifestyle characteristics

Participants self-reported their age (years), smoking habits (never, former, current), education level (no degree or compulsory school, professional school, university), weight (kg), and height (m). Body mass index (BMI) was calculated by dividing weight by the square of height (kg/m²). Additionally, participants responded to the following statements: “I enjoy cooking for others and for myself” and “I buy most or all of my food/groceries myself” using a 7-point Likert scale: strongly disagree, disagree, somewhat disagree, neither agree nor disagree (neutral), somewhat agree, agree, or strongly agree. Responses were grouped into two categories: strongly disagree to neutral and somewhat agree to strongly agree.

### Energy misreporting

Basal metabolic rate (BMR) was calculated with the Schofield equations [25], using age, sex, weight, and height, and converting MJ/24 h to kcal/day. The level of physical activity (PAL) was assessed using the short form of the International Physical Activity Questionnaire (IPAQ) [26, 27]. Using the Guidelines for Data Processing and Analysis [28], IPAQ answers were converted into metabolic equivalent of task (MET)-minutes per week, and participants were classified into low, moderate, or high PAL. Following guidelines by Goldberg et al. [29] and Black [30], we estimated the prevalence of energy misreporting at the individual level for both FFQs by calculating the ratio between EI and BMR (EI: BMR), and assuming coefficients of variation (CV) of 23% for within-subject EI (CV_wEI_), 8.5% for BMR prediction (CV_wB_), and 15% for PAL (CV_tP_), considering 28 days. Participants with low (1.35), moderate (1.55) and high (1.75) PAL were considered energy under-reporters if their EI: BMR ratio was < 0.95, 1.09, or 1.23 and energy over-reporters if their EI: BMR ratio was > 1.93, 2.21, or 2.50, respectively [30]. To align with the objectives of this method‑comparison study, all participants – including those classified as under‑ or over‑reporters by either FFQ – were retained in the analyses, as excluding them would bias the assessment of agreement by removing individuals for whom the FFQs differed most.

### Statistical analyses

In the paper-based FFQ, 0.59% of consumption frequency responses were missing and assumed to be zero (never), while 0.73% of portion sizes were missing and imputed using the reference portion. No missing data were observed in the eFFQ.

For each study participant, we calculated total daily intake of energy and the nutrients available in both FFQs: protein, carbohydrates, dietary fibre, fats, cholesterol, alcohol, vitamin A (retinol and β-carotene), vitamin D, calcium, and iron. Macronutrient intakes were additionally expressed as a percentage of EI (E%). Food items were grouped into 28 food groups common to both FFQs. A few food items exclusive to only one FFQ (e.g., coffee substitute drink, nuts & seeds, plant-based milk substitutes) were excluded from comparisons. Details on food grouping and included and excluded items per food group are provided in Additional file 1. The distribution of nutrient and food group intakes was mostly positively skewed.

Descriptive statistics used to characterise the study participants and their dietary intakes are presented as medians with 25th and 75th percentiles, and as frequencies with percentages. To compare the eFFQ with the paper-based FFQ, the following statistical analyses were performed. We calculated median group-level differences (bias) in estimated nutrient and food group intakes, where differences < 10% were considered good [31, 32]. Consistent with standards in the field, mean differences were also calculated, despite their limited suitability for our data distribution [31, 32]. To assess individual-level differences, we applied the Wilcoxon signed-rank test, with *P* > 0.05 indicating good agreement and *P* ≤ 0.05 indicating poor agreement [31]. The strength and direction of associations between intake estimates from both FFQs were evaluated using Spearman’s correlation coefficient (SCCs) [31, 32] and Lin’s concordance correlation coefficients (LCCs). SCC and LCC values were interpreted as follows: ≥0.50, 0.49–0.20, and < 0.20 indicated a good, acceptable, and poor correlation, respectively [31]. Participants’ intake estimates were also categorised into tertiles to assess classification agreement. We then computed the proportion of participants classified into the same tertiles by both FFQs and those grossly misclassified, i.e., placed in the highest tertile by one FFQ but in the lowest by the other FFQ, or vice-versa [31, 32]. Misclassification of < 10% of participants into opposite tertiles was considered indicative of good agreement at the individual level [31]. Agreement between tertile classifications was further assessed using the weighted Cohen’s kappa statistic (K_w_) [31]. K_w_ values were interpreted as follows: >0.80, 0.80–0.61, 0.60–0.41, 0.40–0.21, and < 0.20 as very good, good, moderate, fair and poor agreement, respectively [33]. Finally, Bland-Altman analysis was performed to calculate the limits of agreement for energy and three macronutrients, with plots used to examine differences and biases between the two FFQs [31, 34]. Additionally, scatter plots were performed. All statistical analyses were performed using STATA software version 16 (StataCorp, College Station, TX, USA).

## Results

### Study participants

Among the 197 volunteers who expressed interest in participating, 162 met the eligibility criteria. To ensure representativeness across sex and age groups, 81 volunteers were invited to take part in the study, while individuals from already complete sex-age strata were not invited. Of those invited, 75 participants (54.7% female; participation rate: 93%) completed both FFQs and were included in the analysis (Table 1). The median (P25-P75) age of the participants was 48 (31–60) years. Most participants were either never smokers (54.7%) or former smokers (37.3%), and a large proportion were highly educated, with 66.7% holding a university degree. The median BMI was 22.6 (20.9–26.1) kg/m². Participants were generally physically active, with a median activity level of 3459 (2038–5226) MET-minutes per week. Notably, a higher proportion of females (92.7%) compared to males (64.7%) were responsible for food shopping. Only a small fraction (4.0%, *n* = 3) adhered to a vegetarian or vegan diet.

**Table 1:** Characteristics of participants who completed both food frequency questionnaires

Characteristics	Overall	Females	Males
Number of participants	75	41	34
Age [years]	48 [31–60]	51 [31–63]	40.5 [32–55]
Age category			
18–37 years	24 (32.0)	12 (29.3)	12 (35.3)
38–57 years	26 (34.7)	12 (29.3)	14 (41.2)
58–75 years	25 (33.3)	17 (41.5)	8 (23.5)
Smoking status			
Current	6 (8.0)	3 (7.3)	3 (8.8)
Former	28 (37.3)	12 (29.3)	16 (47.1)
Never	41 (54.7)	26 (63.4)	15 (44.1)
Education			
No degree / compulsory school	2 (2.7)	1 (2.4)	1 (2.9)
Professional school ^a^	23 (30.7)	14 (34.1)	9 (26.5)
University ^b^	50 (66.7)	26 (63.4)	24 (70.6)
BMI [kg/m^2^]	22.6 [20.9–26.1]	22.5 [20.4–26.8]	23.1 [21–25.3]
BMI category ^c^ [kg/m^2^]			
Underweight	3 (4.0)	3 (7.3)	0 (0)
Normal	48 (64.0)	25 (61.0)	23 (67.6)
Overweight	19 (25.3)	9 (22.0)	10 (29.4)
Obese	5 (6.7)	4 (9.8)	1 (2.9)
BMR ^d^ [kcal/d]	1486 [1335–1663]	1341 [1251–1430]	1665 [1615–1769]
MET ^e^ [min/week]	3459 [2038–5226]	2744 [2040–4980]	4070 [1674–5226]
Enjoy cooking ^f^			
Somewhat agree to strongly agree	60 (80.0)	35 (85.4)	25 (73.5)
Strongly disagree to neutral	15 (20.0)	6 (14.6)	9 (26.5)
In charge of food shopping ^f^			
Somewhat agree to strongly agree	60 (80.0)	38 (92.7)	22 (64.7)
Strongly disagree to neutral	15 (20.0)	3 (7.3)	12 (35.3)
Follow vegetarian/vegan diet ^g^	3 (4.0)	1 (2.4)	2 (5.9)

Data are presented as absolute and relative frequencies, n (%), and as median [25th − 75th percentiles]

*P25* Percentile 25, *P75* Percentile 75, *BMI* Body mass index, *BMR* Basal metabolic rate, *MET* Metabolic equivalent of task

^a^ 3–6 years after compulsory school

^b^ 7 + years after compulsory school

^c^ BMI categorization: < 18.5 = Underweight, 18.5–24.9 = Normal, 25-29.9 = Overweight, ≥ 30 = Obese

^d^ BMR was calculated with the Schofield equations, using age, sex, weight, and height (transformed from MJ/24 h to kcal/day)

^e^ Physical activity level was assessed using the short version of the International Physical Activity Questionnaire (IPAQ). IPAQ answers were converted into MET-minutes per week according to the Guidelines for Data Processing and Analysis

^f^ Responses to the statements “I enjoy cooking for others and for myself” and “I buy most or all of my food/groceries myself”, originally assessed using a 7-point Likert scale, were grouped into: (1) “somewhat agree”, “agree”, and “strongly agree”, and (2) “strongly disagree”, “disagree”, “somewhat disagree”, and “neither agree nor disagree (neutral)”

^g^ Information extracted from the introduction question in the eFFQ

### Estimation of energy misreporting

The mean (SD) EI: BMR ratio was 1.12 (0.42) for the eFFQ and 1.12 (0.52) for the paper-based FFQ. The median (P25-P75) ratio was also similar between FFQs, although the eFFQ (1.02; 0.84–1.40) showed a slightly narrower interquartile range than the paper-based FFQ (1.04; 0.75–1.45). With the eFFQ, 62.7% of participants (*n* = 47) were classified as energy under-reporters, 36.6% as plausible reporters (*n* = 27), and 1.3% as over-reporters (*n* = 1). Similar proportions were observed in the paper-based FFQ: 64.0% (*n* = 48), 32.0% (*n* = 24), and 4.0% (*n* = 3), respectively. Agreement between FFQs was high, with 39 (52.0%) participants classified as energy under-reporters and one (1.3%) as an over-reporter in both FFQs (K_w_=0.52).

### Comparisons at the nutrient level

A comparison of daily energy and 14 nutrient intakes estimated using both the eFFQ and the paper-based FFQ is presented in Table 2. For contextual reference, the corresponding Swiss Dietary Reference Values [35] are also shown. The median (P25-P75) total EI among participants was 1546 (1250–1960) kcal/day for the eFFQ and 1468 (1133–2096) kcal/day for the paper-based FFQ. For most nutrients, including protein, carbohydrates, total sugars, total fat, and SFAs, estimates were also similar between FFQs, with group-level differences < 10%. Exceptions were fibre, polyunsaturated fatty acids (PUFAs), cholesterol, alcohol, calcium, and iron. Notably, the eFFQ estimated a higher median alcohol intake (6.9; 1.9–12.0 g/d) compared to the paper-based FFQ (3.1; 1.2–7.4 g/d). SCCs ranged from 0.45 for SFAs to 0.86 for alcohol, while LCCs ranged from 0.33 for calcium to 0.80 for total sugars. The proportion of participants classified into the same tertile of intake by both FFQs ranged from 40.0% for PUFAs to 73.3% for alcohol. Misclassification into opposite tertiles occurred in fewer than 10% of participants for 14 of the 15 intakes assessed, with SFAs being the exception. Finally, K_w_ values ranged from 0.22 for PUFAs to 0.67 for alcohol, with fair and poor agreement (< 0.40) noted for protein, total fat, SFAs, PUFAs, vitamin A, and calcium. The results presented in Table 2 remained largely unchanged in analyses restricted to the 66 participants who adhered to the predefined FFQ completion order (Additional file 2).

**Table 2:** Comparison of daily energy and nutrient intakes estimated by the eFFQ and the paper-based FFQ (*n* = 75)

Daily intake	Swiss DRV ^a^	eFFQ Median [P25-P75]	Paper-based FFQ Median [P25-P75]	% Group-level differences ^b^	SCC	LCC	% ST ^c^	% ET ^c^	K_w_^d^
Energy (kcal)	♀: 1861–2147 ♂: 2305–2672	1546 [1250–1960]	1468 [1133–2096]	5.3 / *-0.9*	0.67	0.66	61.3	4.0	0.52
Protein (E%)	10% EI ^e^	15.9 [14.6–17.8]	15.3 [13.0–17.3]	4.2 * / *5.0*	0.61	0.56	50.7	5.3	0.39
Carbohydrates (E%)	45–60% EI	39.6 [33.7–43.9]	42.3 [38.0–46.8]	-6.3 * / -6.3	0.69	0.66	62.7	2.7	0.55
Total sugars (disaccharides) (g)	Undefined	78.1 [51.2–88.2]	65.8 [44.7–95.6]	9.1 / *-1.0*	0.75	0.80	53.3	1.3	0.46
Dietary fibre (g)	≥ 30	17.2 [13.6–24.3]	12.5 [9.8–17.0]	37.2 * / *28.6*	0.64	0.50	56.0	4.0	0.46
Total fat (E%)	20–35% EI	38.7 [35.8–42.3]	38.7 [34.2–41.5]	0.0 / *0.3*	0.54	0.56	50.7	9.3	0.34
Saturated fatty acids (E%)	< 10% EI	13.7 [11.3–15.5]	13.3 [12.0–15.8]	2.7 / *-0.5*	0.45	0.52	46.7	13.3	0.25
Monounsaturated fatty acids (E%)	10–15% EI	15.0 [13.1–16.9]	15.3 [13.9–18.5]	-1.9 * / *-6.6*	0.58	0.51	54.7	6.7	0.42
Polyunsaturated fatty acids (E%)	4.5% EI	6.1 [4.8–7.2]	4.8 [4.0–5.9]	27.5 * / *22.5*	0.48	0.40	40.0	9.3	0.22
Cholesterol (mg)	Undefined	222 [175–292]	298 [215–424]	-25.7 * / *-26.7*	0.74	0.53	68.0	5.3	0.58
Alcohol (g)	Undefined	6.9 [1.9–12.0]	3.1 [1.2–7.4]	119.0 * / *50.0*	0.86	0.74	73.3	2.7	0.67
Vitamin A (µg-RE ^f^)	♀: 650 ♂: 750	675 [519–1034]	682 [423–959]	-0.1 * / *4.4*	0.59	0.55	46.7	2.7	0.37
Vitamin D (calciferol, µg)	15	2.6 [2.1–3.2]	2.6 [1.6–3.8]	-0.1 / *-3.3*	0.56	0.37	57.3	8.0	0.43
Calcium (mg)	950	687 [551–884]	792 [597–1288]	-13.3 * / *-21.9*	0.54	0.33	52.0	5.3	0.40
Iron (mg)	♀ and ♂ post-menopause: 11 ♂: 16	7.4 [5.8–9.5]	8.3 [6.4–10.9]	-11.0 * / -14.1	0.57	0.51	58.7	6.7	0.46

*FFQ* Food frequency questionnaire, *DRV* Swiss Dietary Reference Values, *P25* Percentile 25, *P75* Percentile 75, *SCC* Spearman’s rank correlation coefficient (*p* < 0.001 for all nutrients), *LCC* Lin’s concordance correlation coefficient, *ST* Same tertile, *ET* Extreme (opposite) tertile, *K*_w_, Weighted Cohen’s kappa, *E%* Percentage of total energy intake, *EI* Total/daily energy intake, *RE* Retinol equivalent

^a^ Swiss DRVs for male and female adults are defined here: https://www.blv.admin.ch/blv/fr/home/lebensmittel-und-ernaehrung/ernaehrung/empfehlungen-informationen/naehrstoffe/naehrstoffzufuhr-dynamische-tabelle.html

^b^ Group-level differences were based on medians [(median intake eFFQ) / (median intake paper-based FFQ) * 100–100)]. Wilcoxon signed-rank tests were used to assess differences between the two FFQs (*P < 0.05). Group-level mean differences are presented in italic

^c^ Cross-classification analysis results are expressed as a percentage of participants classified into the same and extreme tertiles of distribution by the eFFQ and paper-based FFQ

^d^ Calculation of K_w_ is based on tertiles

^e^ DRV: 0.83 g/kg of body weight. This is equivalent to 49.8 g for a female of 60 kg (= 10.0% of TEI of 2000 kcal) or to 62.3 g for a male of 75 kg (10.3% of 2,400 kcal)

^f^ 1 µg RE equals 1 µg of retinol + 12 µg of β-carotene for the paper-based FFQ and 1 µg of retinol + 6 µg of β-carotene + 12 µg of other provitamin A carotenoids for the eFFQ

**Fig. 1:**
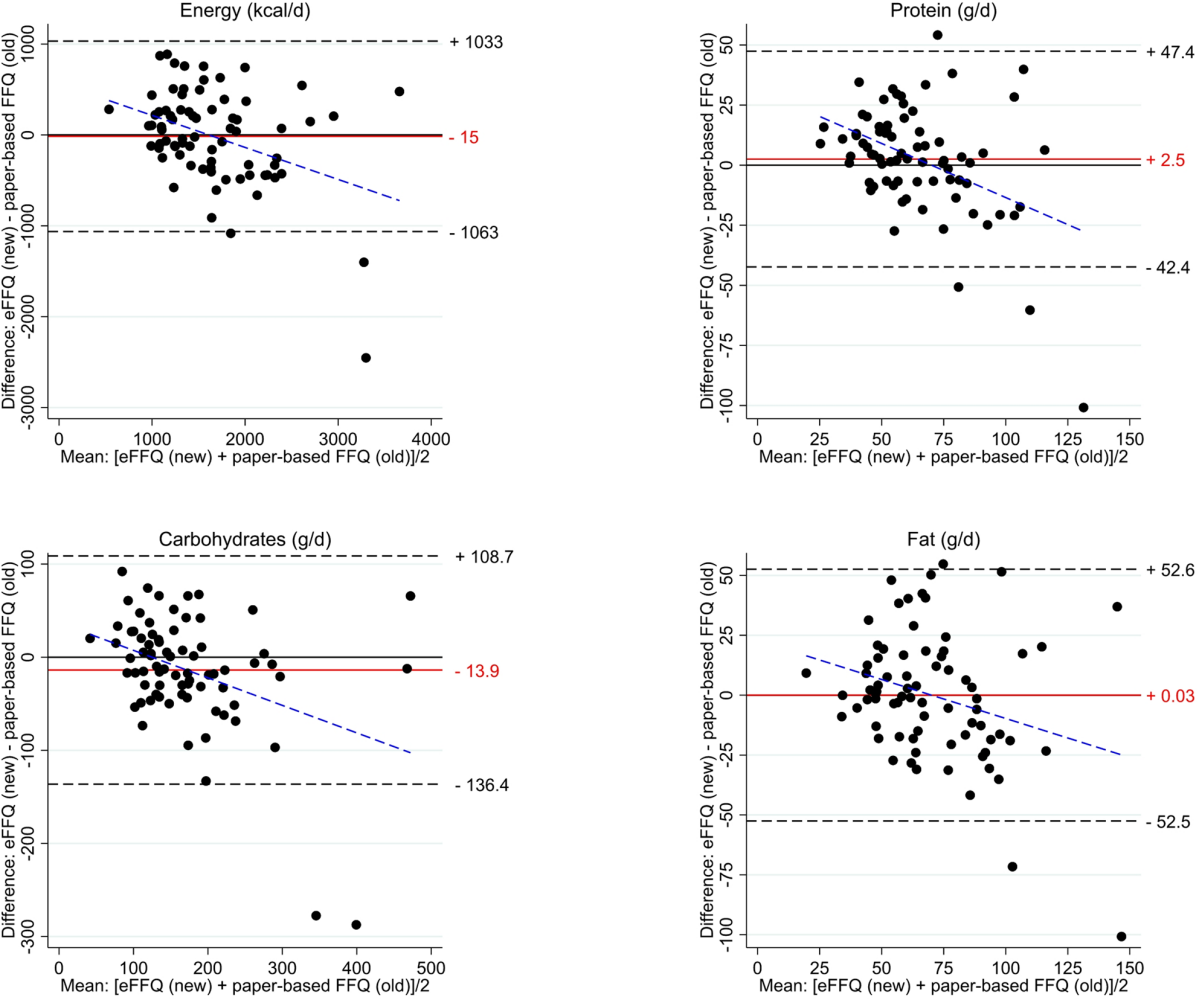
Bland-Altman plots of the differences in energy and three macronutrient intakes estimated by the eFFQ and paper-based FFQ (*n* = 75). Solid red horizontal lines represent the mean difference and the dashed black lines 95% limits of agreement. The dashed blue line represents the regression of the differences on the mean, indicating proportional bias. FFQ, Food Frequency Questionnaire

Figure 1 displays Bland-Altman plots comparing EI (kcal/d) and absolute intakes (g/d, not E%) of carbohydrates, protein, and total fat between the eFFQ and the paper-based FFQ. Overall, the mean differences between FFQs were small, indicating a good agreement at a group level. However, the 95% limits of agreement were wide (2096 kcal/day for energy; 89.8 g/day for protein; 245.1 g/day for carbohydrates; 105.1 g/day for total fat), reflecting substantial variability at the individual level. Differences between the two FFQs increased at higher intake levels, suggesting proportional bias. Of note, the paper-based FFQ estimated substantially higher intakes for two participants (i.e., EI > 3500 kcal). These extreme values were not observed in the eFFQ and contributed to the wide limits of agreement. This pattern of greater variability at higher intake levels, driven by a few high-intake outliers, is also visible in the scatter plots (Additional file 3), with SCCs for energy and macronutrients ranging from 0.53 to 0.74 (*p* < 0.001).

An effect regarding order of FFQ completion was observed: participants who completed the eFFQ first showed higher correlations for energy (0.72 vs. 0.60), protein (0.72 vs. 0.63), and carbohydrate (0.74 vs. 0.69) than those who completed the paper-based FFQ first. Correlations for total fat were slightly lower among those completing the eFFQ first (0.52 vs. 0.58).

### Comparisons at the food group level

Table 3 presents a comparison of daily intakes of the 28 food groups estimated using both the eFFQ and the paper-based FFQ. The estimated median intakes were significantly higher in the eFFQ for water; potatoes & potato products; milk; red meat; vegetable oil; added sugars, jam & honey; cakes, pastries, desserts & ice-creams; biscuits & confectionaries; sugar-sweetened beverages; beer; wine & other alcohols; and mixed dishes & soups, and particularly for plant-based protein-rich foods and cream, fatty sauces & other fats. By contrast, the estimated intakes were significantly lower in the eFFQ for vegetables; cheese; fish & seafood; and eggs. SCCs ranged from 0.33 (cream, fatty sauces & other fats) to 0.91 (beer), but SCC for water was 0.07. LCCs ranged from 0.06 (cream, fatty sauces & other fats) to 0.82 (wine & other alcohols). The percentage of participants classified in the same tertile ranged from 52.0% (cream, fatty sauces & other fats) to 84.0% (beer), with an extreme value at 22.7% for water. Small differences at the individual level (i.e., ≤ 10% of participants classified in the opposite tertile by both FFQs) were observed for 24 of the 28 food groups. K_w_ values ranged from 0.31 (cream, fatty sauces & other fats) to 0.80 (beer), with an extreme value at -0.03 for water. Fair and poor agreements (< 0.40) were additionally observed for vegetable oil and mixed dishes & soups.

**Table 3:** Comparison of daily food group intakes estimated by the eFFQ and the paper-based FFQ (*n* = 75)

Daily food group intake ^a^	eFFQ Median [P25-P75]	Paper-based FFQ Median [P25-P75]	% Group-level differences ^b^	SCC	LCC	% ST ^c^	% ET ^c^	K_w_^d^
Water (mL)	1350 [750–1800]	1050 [717.9–1076.8]	28.6 * / *34.2*	0.07	0.16	22.7	16.0	-0.03
Tea (mL)	235.7 [64.3–300.0]	200.0 [42.9–700.0]	17.9 / *-10.7*	0.87	0.79	76.0	0.0	0.71
Coffee (mL)	125.0 [41.7–312.5]	120.0 [40.0–320.0]	4.2 / *2.1*	0.76	0.68	62.7	2.7	0.51
Vegetables (g)	116.5 [88.4–168.5]	133.8 [93.6–204.5]	-12.9 * / *-14.4*	0.66	0.51	61.3	4.0	0.52
Fruit (g)	155.0 [84.0–253.0]	148.0 [78.4–266.8]	4.7 / *-3.6*	0.76	0.68	66.7	4.0	0.58
Fruit juices (mL)	17.9 [0–42.9]	14.3 [0–60.7]	25.0 / *-10.7*	0.85	0.72	80.0	2.7	0.75
Breakfast cereals (g)	3.6 [0–20.0]	2.7 [0–15.0]	33.3 / *8.4*	0.66	0.29	62.7	2.7	0.55
Potatoes & potato products (g)	28.0 [16.1–45.6]	21.4 [14.5–32.1]	30.5 * / *20.0*	0.57	0.45	54.7	6.7	0.41
Bread products (g)	53.2 [28.7–71.8]	50.0 [21.4–78.9]	6.4 / *-3.6*	0.78	0.57	61.3	4.0	0.52
Pasta, rice, polenta, couscous & other grains (g)	84.4 [56.4–119.0]	83.6 [37.1–126.4]	1.0 / *-10.6*	0.64	0.51	53.3	6.7	0.40
Milk (mL)	20.8 [1.5–78.1]	9.6 [0.0–45.0]	116.0 * / *35.8*	0.74	0.32	58.7	5.3	0.49
Yogurt & fresh cheese (g)	38.6 [16.1–90.0]	38.6 [8.0–97.5]	0.0 / *-10.3*	0.85	0.81	68.0	2.7	0.60
Cheese (g)	30.3 [14.2–47.4]	40.7 [25.7–90.0]	-25.7 * / *-37.1*	0.71	0.37	62.7	2.7	0.55
Red meat (g)	45.8 [26.0–72.9]	30.1 [20.0–56.2]	51.2 * / *14.3*	0.71	0.57	62.7	2.7	0.55
Poultry (g)	28.1 [11.7–28.1]	18.8 [8.9–32.1]	49.9 / *6.9*	0.63	0.70	64.0	5.3	0.51
Fish & seafood (g)	11.8 [9.3–24.9]	26.8 [10.0–35.7]	-55.8 * / *-36.4*	0.62	0.39	58.7	9.3	0.44
Eggs (g)	11.1 [4.6–26.0]	21.4 [10.7–25.0]	-48.1 * / *-34.0*	0.74	0.69	60.0	12.0	0.47
Plant-based protein-rich foods (incl. legumes, tofu) (g)	11.0 [2.9–26.6]	0 [0–8.9]	NA / *239.8*	0.64	0.48	68.0	6.7	0.58
Vegetable oil (mL)	11.6 [9.3–16.6]	10.2 [6.8–16.3]	13.8 * / *14.5*	0.42	0.29	53.3	10.7	0.35
Butter & margarine (g)	5.2 [0.9–10.4]	4.0 [0.9–9.6]	30.0 / *-14.9*	0.82	0.74	66.7	2.7	0.62
Cream, fatty sauces & other fats (g)	9.9 [4.5–17.9]	0.9 [0–2.1]	1012.8 * / *244.7*	0.33	0.06	52.0	13.3	0.31
Added sugars, jam & honey (g)	13.0 [2.3–26.0]	10.0 [1.8–20.0]	30.0 * / *18.5*	0.79	0.76	73.3	4.0	0.65
Cakes, pastries, desserts & ice-creams (g)	23.3 [13.1–35.9]	17.5 [8.0–35.4]	33.2 * / *0.0*	0.64	0.46	60.0	2.7	0.52
Biscuits & confectionaries, incl. chocolate (g)	12.7 [6.5–23.6]	8.5 [3.8–15.9]	48.3 * / *23.4*	0.75	0.73	62.7	0.0	0.58
Sugar-sweetened beverages (mL)	10.0 [0–33.5]	7.1 [0–26.8]	40.6 * / *30.6*	0.65	0.57	70.7	8.0	0.60
Beer (mL)	44.6 [0–107.1]	26.8 [0–75.0]	66.7 * / *49.0*	0.91	0.74	84.0	0.0	0.80
Wine & other alcohols (mL)	31.3 [7.1–51.2]	19.6 [5.4–35.7]	59.4 * / *34.1*	0.88	0.82	70.7	4.0	0.64
Mixed dishes & soups (g)	76.4 [43.9–113.2]	53.1 [33.5–96.4]	43.8 * / *14.9*	0.47	0.57	53.3	8.0	0.39

*FFQ* Food frequency questionnaire, *P25* Percentile 25, *P75* Percentile 75, *SCC* Spearman’s rank correlation coefficient (*p* < 0.001 for all foods groups, except for water *P* = 0.52), *LCC* Lin’s concordance correlation coefficient, *SQ* Same tertile, *EQ* Extreme (opposite) tertile, *K*_w_, Weighted Cohen’s kappa, *NA* Not applicable as median = 0 for the paper-based FFQ

^a^ Foods included in each food group are described in Additional file 1. Food groups were defined based on the levels of the Swiss food-based dietary guidelines (Swiss Food Pyramid): https://www.blv.admin.ch/blv/en/home/lebensmittel-und-ernaehrung/ernaehrung/empfehlungen-informationen/schweizer-ernaehrungsempfehlungen.html

^b^ Group-level differences were based on medians [(median intake eFFQ) / (median intake paper-based FFQ) * 100–100)]. Wilcoxon signed-rank tests were used to assess differences between the two FFQs (*P < 0.05). Group-level mean differences are presented in italic

^c^ Cross-classification analysis results are expressed as a percentage of participants classified in the same and extreme tertiles of distribution by the new eFFQ and the paper-based FFQ

^d^ Calculation of K_w_ is based on tertiles

## Discussion

Variations in absolute energy, nutrient, and food group intakes between the recently developed eFFQ and the old paper-based FFQ were expected and documenting them is an essential first step towards understanding methodological differences that may affect comparisons of dietary intake over time (e.g., trend analyses) and across populations. Both FFQs underestimated EI and produced broadly comparable absolute estimations at the nutrient level, except for alcohol. As expected, differences at the food group level were observed for several food groups, notably due to the updated food list of the eFFQ. Agreement in classifying participants between the two FFQs was good (SCCs > 0.50 and K_w_ >0.40) for most nutrients (except SFAs and PUFAs) and food groups (except water; vegetable oils; cream, fatty sauces & other fats; and mixed dishes & soups), highlighting the complexity of assessing these items in FFQs. Overall, both FFQs similarly ranked participants. These findings indicate that, although absolute intakes may differ between the two FFQs, they performed similarly in ranking or clustering individuals based on usual dietary habits, a key objective of FFQs in nutritional epidemiology [15, 36].

### Energy misreporting

Both FFQs underestimated EI and EI: BMR, with approximately 60% of participants classified as under-reporters. Energy under-reporting is common in self-reported dietary assessment methods, including FFQs [37, 38]. In adults, underestimation between reported EI and actual energy expenditure, measured using doubly labelled water, can reach up to 32% [37], notably because FFQs present a definite, non-exhaustive list of foods. Of note, both FFQs were designed to capture more than 90% of total EI despite containing fewer than 100 items.

The median (P25-P75) EI reported using the eFFQ was 1546 (1250–1960) kcal/day. In the previous Swiss eFFQ validation study, including a larger sample with German-speakers, median EI was 1638 (1357–2003) kcal/day, whereas the reference method (4-day food records) estimated a median EI of 2023 (1715–2349) kcal/day. This consistent underestimation may partly reflect the use of a single standard portion sizes in the eFFQ, which limits the ability to capture individual variation.

Although average differences between the eFFQ and the paper-based FFQ for energy and macronutrient intakes were small (Fig. 1), the limits of agreement were wide, indicating that discrepancies at the individual level can be substantial and limit the interchangeability of the two FFQs for individual dietary assessment. This is illustrated by two participants – a male and a female – who reported exceptionally high physical activity levels (> 10,000 MET-minutes per week) and had markedly higher estimated intakes captured only by the paper‑based FFQ. If participants habitually consume larger-than-standard portions, the paper-based FFQ can reflect this by allowing a “larger” portion selection, whereas the eFFQ cannot, owing to its standard portion size. Applying a single standard portion size may also contribute to greater under-reporting in samples with high PAL, such as ours (median close to 3500 MET-min/week). Individuals with higher PAL are likely to consume larger portion sizes than those in the population-based menuCH sample from which standard portion sizes were derived for the eFFQ. In addition, the source dataset used for eFFQ development already included 17% of participants classified as under‑reporters [23], suggesting that some residual bias may have been incorporated into the eFFQ. Finally, although the eFFQ allows participants who identify as vegetarian or vegan to skip certain food‑group questions (e.g., meat, dairy) – a feature our team has previously found can lead to occasional omission of foods still consumed – its impact in the present study was likely minimal, as only 4% of participants used this option. Overall, these factors are likely to have contributed to the high prevalence of under‑reporting identified with the eFFQ.

In the present study, the EI estimated by the paper-based FFQ was even slightly lower (1468 kcal/day) than that of the eFFQ. For comparison, the Bus Santé study 1993–1994 reported higher EI in the Geneva population aged 35 to 74 years in (*n* = 1489), with median intakes for males between 2169 and 2390 kcal/day and for females between 1897 and 1907 kcal/day, depending on age groups [8]. In 2009–2012, the CoLaus|PsyCoLaus study found an EI below 1800 kcal/day for both males and females aged 40 to 80 years living in Lausanne (*n* = 3866) [39]. This progressive decline in estimated EI suggests that the food list of the paper-based FFQ, developed in the 1990s, may have become increasingly outdated and less able to capture contemporary dietary patterns. For example, there was a limited number of items for assessing convenience and ready-to-eat foods (Additional file 1). Such omissions may contribute to greater under-reporting over time. Under-reporting in the general population might also have increased in recent years, possibly linked to rising obesity rates or increased social desirability bias, both associated with higher under-reporting [40, 41]. Finally, another broader explanation for the high prevalence of energy under‑reporting in both FFQs is that our study population had different dietary habits compared to the general French-speaking population.

### Comparisons at the nutrient level

At a group level, the eFFQ and paper-based FFQ produced broadly similar estimates of total daily energy and most nutrient intakes. Variations in central tendencies were generally under 10%, although differences existed in the design of the FFQs, including the food composition databases used for linkage with food items (Swiss and German vs. French and Swiss, respectively). Intakes were higher in the eFFQ for fibre, PUFAs, and alcohol, lower for cholesterol, and slightly lower for calcium and iron. For fibre, the median intake was 17.2 g/d (i.e., 11.1 g/1000 kcal/d) with the eFFQ and 12.5 g/d (i.e., 8.5 g/1000 kcal/d) with the paper-based FFQ. Knowing that menuCH estimated a median intake of 18.7 g/d (i.e., 8.9 g/1000 kcal/d) among adults aged 18 to 75 years [42], the higher intake reported with the eFFQ is probably closer to the true intake. For PUFAs, the median intake in menuCH was 7.4 g/d (3.2% of EI) [42], compared to 4.8% of EI with the paper-based FFQ and 6.1% with the eFFQ. Estimating PUFAs intake with FFQs is challenging, as oils and fats are often used for cooking and consumed within mixed dishes and sauces, which are not always correctly reported by participants [13, 43–45]. In addition, PUFAs were also probably underestimated in the paper-based FFQ, as no food item was related to nuts and seeds. Finally, the eFFQ estimated alcohol intake (6.1 g/d) compared to the paper-based FFQ (3.1 g/d). As the FFQs had four very similar items to assess alcoholic beverages, portion sizes very likely played a role in this difference. For instance, the standard portion sizes for beer and wine were 500 ml and 200 ml in the eFFQ, while the reference portion sizes were 300 ml and 150 ml in the paper-based FFQ, respectively. These discrepancies reflect the fact that the Swiss eFFQ was developed for both German- and French-speaking regions, whereas the paper-based FFQ was specifically tailored to the French-speaking part of Switzerland, where beer and wine portion sizes are smaller.

### Comparisons at the food group level

Overall, median food group intakes assessed by both FFQs were comparable to those found in menuCH [23], except for milk and sugar-sweetened beverages, which were lower in both FFQs. No comparison with menuCH was, however, possible for breakfast cereals; eggs; and mixed dishes & soups. In our study, food groups that are consumed daily and in larger quantities (e.g., tea, coffee, bread, and grain products) or in standardized portion sizes (e.g., cups of yogurt, pieces of fruit) have shown less group-level bias and higher correlations and ranking abilities between both FFQs. This is consistent with findings reported in the literature [13, 43, 44].

Median intakes reported with the eFFQ were higher for several food groups compared to the paper-based FFQ, particularly plant-based protein-rich foods; cream, fatty sauces & other fats; mixed dishes & soups; and beverages. These differences were expected, given that the eFFQ includes a more detailed and updated food list (Additional file 1). The broader food coverage in the eFFQ likely facilitated more complete dietary recall, prompting participants to report foods that may have been overlooked in the paper-based FFQ. The higher SCCs observed among participants completing the eFFQ first further suggest a potential learning or priming effect. The eFFQ, for example, includes more items for legumes and other plant-based proteins (3 vs. 1 items); cream, fatty sauces & other fats (6 vs. 3 items); and mixed dishes & soups (8 vs. 4 items). In the case of sauces, the paper-based FFQ included only “mayonnaise”, while the eFFQ explicitly queried a wider variety of sauces, including those typically consumed warm with meat or pasta. As previously discussed, estimating oils, fats, and sauces with accuracy using FFQs is challenging [13, 43, 44]. Similarly, the eFFQ validation study reported limited agreement with a 4-day food record for these food items [13].

For beverages, such as water, milk (including in coffee), sugar-sweetened, and alcoholic beverages, discrepancies between the two FFQs could arise from differences in the highest frequency category allowed (≥ 5 in the eFFQ vs. ≥2 in the paper-based FFQ) and in portion size definitions. Water intake was measured very inconsistently by both FFQs (SCC ≈ 0.07). The limited frequency range in the paper-based FFQ (maximum ≥2 x/day, coded as 3.5 x/day [18]) may have underestimated intake, whereas the eFFQ allowed for higher reporting frequencies (≥ 5 x/day). Portion sizes were also different: 200 ml vs. 300 ml, respectively. For sugar-sweetened beverages, the reference portion size in the paper-based FFQ (200 ml) was almost half that of the standard portion size in the eFFQ (375 ml). Differences in frequencies and portion sizes can lead to large biases in total intake estimates, especially for water and other frequently consumed items. Additionally, beverages were assessed at the end of each FFQ, so participants might have overlooked these items due to fatigue, particularly if completing both FFQs on the same day. Of note, the median (P25-P75) time to complete the eFFQ was 34 (24–46) minutes [13] (no data available for the paper version).

### Strengths and limitations

Several limitations should be considered when interpreting our findings. First, we employed a convenience sample of highly educated, health-conscious adults, mostly engaged in cooking and grocery shopping, and with BMI values predominantly within the normal range according to WHO classifications. While this does not affect the within-person methodological comparison per se, it may limit the applicability of the observed differences between the paper-based and electronic FFQs to populations with lower digital literacy or different response behaviours. Second, we observed a small FFQ completion order effect, whereby participants who completed the eFFQ first showed higher correlation coefficients for intakes of energy, protein and carbohydrates, consistent with a learning effect [15], which may have influenced comparisons. The short one‑week interval between administrations was chosen to ensure that both FFQs captured dietary intake over a comparable period (i.e., the preceding four weeks). Although carry‑over effects cannot be fully excluded, participants reported stable weight and dietary habits. Thus, the observed differences between FFQs likely reflect a combination of methodological differences and minor behavioural changes related to the repeated administration, rather than substantial variation in actual intake. Third, differences in questionnaire format (electronic vs. paper) could themselves influence reporting [16]. The eFFQ, being electronic and forcing an answer, ensured all questions were answered, whereas a few items were missing in the paper-based FFQ. The missing data in the paper‑based FFQ might have been avoided with a protocol‑defined completeness check by study staff and prior agreement with participants to permit follow‑up for retrieving missing information. Lastly, our comparison was limited to nutrients estimated by both FFQs, preventing a more complete evaluation of micronutrient intakes. Despite these limitations, our study met the a priori sample size requirement for method comparison and applied multiple statistical approaches to assess group-level bias and agreement. We also explicitly documented content differences between the two FFQs, which is a strength that aids the interpretation of the results.

### Implications for practice and future development

Currently, the Swiss eFFQ is being fine-tuned to be applied in different research settings. For the eFFQ, enhancements, such as visualisation of adaptive portions or better guidance for reporting oils, fats and sauces, could improve the accuracy of future FFQs developed in Switzerland. Regarding the features that allow participants to skip food items based on reporting being vegetarian or vegan, further research should explore strategies to balance accurate dietary assessment with usability, ensuring that respondent engagement is maintained without compromising data quality. Future research should also evaluate how nutrient and food group intakes estimated by the eFFQ compare with the population’s usual intake, using methods that account for intra- and inter-individual variability in the dietary intake. This would strengthen the evidence for using the eFFQ in population monitoring and dietary guideline adherence assessments.

Although EI is often reported with FFQs, for population surveillance and monitoring, such as assessing compliance with dietary guidelines, researchers and policy makers are primarily interested in nutrient and food group intakes to study trends over time or compare populations. Consequently, discrepancies between the electronic and paper-based FFQs for nutrients and food groups are of greater relevance than differences in EI alone. In our study, most nutrients (except SFAs and PUFAs) and food groups (except water; vegetable oils; cream, fatty sauces & other fats; and mixed dishes & soups) presented acceptable agreement between the two FFQs, suggesting that data for these components can reasonably be compared directly across tools. For other nutrients and food groups, researchers and policy makers analysing trends over time or comparing populations should consider applying adjustment or calibration between the two FFQs or interpret the results with caution by taking into account the methodological variations (e.g., number of food items in the food list, standard portion sizes) rather than attributing discrepancies to true changes in intake.

For epidemiological research investigating associations between dietary intake and disease, as previously conducted with the paper-based FFQ [39, 46–48], the good agreement in ranking individuals is reassuring and consistent with findings from the eFFQ validation study [13]. Therefore, association studies can rely on the eFFQ to classify individuals’ diets similarly to the old paper-based FFQ, with the exception of the nutrients and food groups mentioned above. For these components, the same precautions regarding calibration or careful interpretation should be applied before conducting analyses or drawing conclusions.

As dietary habits have evolved considerably since the 1990s [9, 10], the use of the newly developed eFFQ based on menuCH data is advised to more accurately capture dietary habits. In addition to an updated food list better aligned with the current Swiss diet, the eFFQ offers further advantages. It provides estimated daily intakes for more nutrients – specifically energy and all 42 nutrients available in the Swiss food composition database [22] – while its digital format reduces the burden on researchers by streamlining data processing and quality [49–51]. Finally, the eFFQ can be more easily implemented in digital cohorts or web-based health platforms across the country, as it is available in both German and French.

## Conclusions

Our study compared dietary intake estimates obtained from the newly developed and validated eFFQ with those from the old paper‑based FFQ. Overall, both FFQs ranked individuals similarly, supporting the use of the eFFQ in future studies among French-speaking adults living in Switzerland. The updated food list and digital format of the eFFQ provide important practical advantages and better reflect contemporary dietary habits. Individual-level differences were documented, and group‑level differences were observed for some nutrients (i.e., SFAs and PUFAs) and most food groups (especially water; vegetable oils; cream, fatty sauces & other fats; and mixed dishes & soups). Therefore, researchers and policy makers transitioning from the paper‑based FFQ to the eFFQ or comparing data from both FFQs should account for these discrepancies to ensure valid interpretation of dietary intake estimates.

## Supplementary Information

Supplementary Material 1.

## Data Availability

Data described in the manuscript will be provided by the corresponding author upon reasonable request.

## Abbreviations

BMR: Basal Metabolic Rate
BMI: Body Mass Index
CV: Coefficient of Variation
E%: Percentage of Energy Intake
EI: Energy Intake
EI:BMR: Energy Intake-to-Basal Metabolic Rate ratio
IPAQ: International Physical Activity Questionnaire
PAL: Level of Physical Activity
FFQ: Food Frequency Questionnaire
LCC: Lin’s Concordance Correlation Coefficient
PUFAs: Polyunsaturated Fatty Acids
SFAs: Saturated Fatty Acids
SCC: Spearman’s Correlation Coefficient
eFFQ: Swiss Electronic Food Frequency Questionnaire
menuCH: Swiss National Nutrition Survey
