# Supplementary material for: Agreement between a new web-based and a legacy paper-based food frequency questionnaires in French-speaking Switzerland
Source: Nutr J. 2026 Mar 27;25:59. doi: 10.1186/s12937-026-01309-7 (PMC13147631; doi:10.1186/s12937-026-01309-7)
Supplement: Supplementary file 1 — Supplementary Material 1. [file 12937_2026_1309_MOESM1_ESM.docx]

**ADDITIONAL FILES**

**Agreement between a new web-based and a legacy paper-based food frequency questionnaire in French-speaking Switzerland**

Angeline Chatelan *et al.*

**Additional file 1: List of food items included in the new eFFQ and the old paper-based FFQ, by food group.**

| **Food** **groups ^a^** | **Items in the eFFQ (number of items per food group)** | **Items in the paper-based FFQ (number of items per food group)** |
| --- | --- | --- |
| **Water** | - Water (**1**). | - Mineral water (Aproz^®^, Valser^®^, San Pellegrino^®^); - Mineral water (Perrier^®^, Vittel^®^, Volvic^®^); - Tap water, mineral water (Henniez^®^, Evian^®^, Vichy^®^) (**3**). |
| **Tea** | - Tea (e.g. fruit, black, herbal tea) (**1**). | - Tea, herbal tea (**1**). |
| **Coffee** | - Espresso or ristretto; - Coffee (black/without milk); - Latte or coffee with milk/cream (conversion factor: *0.5); - Cappuccino (conversion factor: *0.5); - Latte macchiato (conversion factor: *0.33) (**5**). | - Coffee (**1**). |
| **Vegetables** | - Green peas or green beans; - Carrots or beetroot; - Fennel, asparagus, chard, or artichokes; - Broccoli, cauliflower, or other cabbage vegetables; - Spinach (leaf or creamed spinach); - Mushrooms (champignon); - Tomato, cucumber, or peppers (as raw or cooked vegetables); - Zucchini, eggplant or pumpkin; - Salad (leaf, iceberg, lettuce, lamb's lettuce); - Onions, leeks, or garlic; - Avocado (incl. guacamole); - Tomato sauce (**12**). | - Green beans, spinach; - Cauliflower, broccoli; - Tomatoes; - Carrots; - Green salad; - Green peas, sweetcorn; - Avocado; - Tomato sauce (**8**). |
| **Fruit** | - Grapes or berries (strawberries, blueberries, raspberries); - Apricot, peach, or nectarine; - Tangerine or orange; - Tropical fruits (banana, kiwi, fig, pineapple, mango); - Apple or pear; - Fruit puree or compote (apple, plums); - Dried fruit (dates, raisins, or apricots) (**7**). | - Banana, apple, pear, plum, grapes; - Citrus fruits (orange, tangerine); - Peach, apricot, melon; - Berries (strawberries, blueberries); - Kiwi; - Fruit, preserve or puree (**6**). |
| **Fruit juices** | - Fruit juice (orange, apple, multifruit, fresh or bottled) (**1**). | - Fresh fruit juice; - Bottled fruit juice (**2**). |
| **Breakfast cereals** | - Cornflakes, crunchy muesli, and sweetened breakfast cereals; - Unsweetened flakes (oat flakes) (**2**). | - Muesli (cereals mixes); - Cornflakes, puffed rice (**2**). |
| **Potatoes& potato products** | - French fries or croquettes; - Roesti or potato gratin; - Boiled potatoes or gnocchi (**3**). | - Potatoes, boiled; - French fries (**2**). |
| **Bread products** | - Wheat, white or toast bread (incl. rolls); - Semi-white or spelt bread; - Wholegrain or seeded bread; - Plaited bread or croissants (butter, pretzel or wholegrain); - Rusks or crispbread (DAR-VIDA^®^, Blevita^®^, rice cakes/papers) (**5**). | - White or toast bread; - Wholegrain bread, rye bread; - Rusks, Swedish bread (**3**). |
| **Pasta, rice, polenta, couscous & other grain products** | - Corn, semolina, couscous, or quinoa; - Rice or rice dishes; - Pasta or pasta dishes (**3**). | - Pasta; - Ravioli, tortellini, cannelloni; - Rice; - Couscous, semolina (**4**). |
| **Milk (including in coffee)** | - Milk in latte or coffee with milk/cream (conversion factor: *0.5); - Milk in cappuccino (conversion factor: *0.5); - Milk in latte macchiato (conversion factor: *0.67); - Whole milk (as a drink); - Semi-skimmed milk as a drink (**5**, but **2** **new** as 3 items already included in coffee). | - Skimmed milk in coffee; - Semi-skimmed or whole milk in coffee; - Skimmed milk as a drink; - Semi-skimmed or whole milk as a drink (**4**). |
| **Yogurt & fresh cheese** | - Full-fat yoghurt, plain; - Full-fat yogurt, fruit, and flavouring; - Low-fat yoghurt (light), plain; - Low-fat yogurt (light), fruit and flavourings (**4**). | - Plain yogurt; - Low-fat yogurt; - Fruit/aroma yogurt; - Low-fat quark (**4**). |
| **Cheese** | - Hard, sliced or grated cheese (e.g. Gruyère, Parmesan, Emmental, excluding raclette and fondue cheese); - Raclette or cheese fondue; - Mozzarella, cream cheese, quark or cottage cheese; - Soft cheese (e.g. Brie, Camembert, Tomme, Gorgonzola) (**4**). | - Cottage cheese, ricotta; - Feta, mozzarella; - Gruyère, Tomme, Camembert; - Cheese fondue (**4**). |
| **Red meat** | - Salami, salametti or landjäger; - Ham, dried meat, or cold cuts; - Lamb or veal (fillet, cutlet); - Game, horse, or goat meat; - Minced meat or stew; - Pork (steak, roast, cutlet); - Beef (sliced, steak, entrecôte, fillet); - Sausage or meat loaf; - Offal (liver or tongue) (**9**). | - Beef, horse, veal (steaks, lean meat); - Hamburger, rib steak, roasted meat (beef, horse, or veal); - Cured ham, mutton, or pork chops; - Sausage, salami, ham; - Pâté, terrine; - Cervelas, wieners; - Frankfurter, small sausages; - Liver (veal or pork), offal (**8**). |
| **Poultry** | - Chicken meat (breast, thigh, sliced chicken) (**1**). | - Chicken, skinned; - Chicken, with skin; - Liver (poultry) (**3**). |
| **Fish & seafood** | - Salmon, mackerel, or herring; - Tuna, cod, trout, or perch; - Fish crispies or fish fingers; - Seafood or shellfish (shrimps or mussels) (**4**). | - Salmon (fresh or smoked); - Fried or breaded fish; - Tuna in oil; - White fish (cod, trout, hake); - Seafood (shrimps, mussels) (**5**). |
| **Eggs** | - Eggs (boiled egg, scrambled egg, or fried egg) (**1**). | - Eggs (**1**). |
| **Plant-based protein-rich foods (including legumes, tofu)** | - Tofu or soy (soy granules); - Meat substitute products (burgers, nuggets, sausages, or veggie steak, from Beyond Meat^®^, Planted^®^ or Garden Gourmet^®^); - Pulses (lentils, chickpeas, beans) (**3**). | - Tofu (**1**). |
| **Vegetable oil** | - Salad dressing (French, Italian, yoghurt, vinaigrette, or balsamic vinegar) (conversion factor: *0.5); - Olive oil; - Rapeseed oil; - Sunflower oil; - Other vegetable oils (**5**). | - Vinaigrette sauce (conversion factor: *0.5); - Olive oil; - Peanut oil; - Sunflower oil (**4**). |
| **Butter & margarine** | - Butter, margarine, or other fats (vegetable or animal) (**1**). | - Margarine, low fat (as spread on bread); - Butter (as spread on bread); - Butter (for cooking); - Margarine (for cooking) (**4**). |
| **Cream, fatty sauces & other fats** | - Cold sauces (e.g. mayonnaise, ketchup, pesto); - Meat, cream, or gravy sauces; - Hot sauces (e.g. béchamel, carbonara, curry or cheese sauces); - Whole cream; - Semi-skimmed cream/reduced-fat cream; - Soy cream and other plant-based cream substitutes (**6**). | - Cream (35% fat); - Mayonnaise; - Coffee creamer (**3**). |
| **Added sugars, jam & honey** | - Chocolate spread (e.g. Nutella^®^); - Honey; - Jam; - Sugar (e.g. white, brown) (**4**). | - Honey, jam; - Sugar (**2**). |
| **Cakes, pastries, desserts & ice-creams** | - Fruit tart (e.g. apple, plums, rhubarb, cherries); - Cake or tart; - Sweet pastries (e.g. Danish pastries, almond, chocolate or nut croissants); - Dessert (e.g. cream, mousse, tiramisu, caramel cake, pudding) or ice cream (**4**). | - (Chocolate) croissant; - Fruit tart; - Cream tart, cream cake; - Cake, dried pastries; - Ice cream, sorbet (**5**). |
| **Biscuits and confectionaries (including chocolate)** | - Biscuits; - Chocolate (including chocolate sticks, bars) or pralines; - Sweets (e.g. gummy bears, sweets or chewing gum) (**3**). | - Biscuits, cookies; - Chocolate (**2**). |
| **Sugar-sweetened beverages** | - Sugary drinks (e.g. Coca Cola^®^, Rivella red^®^, iced tea, syrup) (**1**). | - Lemonade, soda, syrup (**1**). |
| **Beer** | - Beer (**1**). | - Beer (**1**). |
| **Wine & other alcohols** | - Sparkling wine or champagne; - Wine; - Spirits or liqueurs (**3**). | - Wine, champagne; - Aperitifs (Martini^®^); - Spirits (whisky, liquor) (**3**). |
| **Mixed dishes & soups** | - Vegetable and potato soups or stews; - Cream soups (e.g. vegetable, pumpkin, asparagus, or tomato cream soup); - Bouillons or clear soups (with or without pasta); - Meat and fish soups or stews; - Pizza; - Sandwiches or filled savoury croissants (e.g. with ham or cheese); - Savory pastry (e.g. cheese, spinach) or tarte flambée; - Kebabs or burgers (**8**). | - Vegetable broth; - Vegetable soup (peas, beans, minestrone); - Pizza; - Quiche Loraine (**4**). |
| **Excluded from analysis at the food group level** | - Coffee substitute drink (e.g. made from chicory); - Soy drink, oat drink and other plant-based milk substitutes; - Soy yogurt or other plant-based yogurt alternatives, plain; - Soy yogurt or other plant-based yogurt alternatives, fruit and flavourings; - Nuts or seeds; - Low-sugar, light or zero drinks (e.g. Coca Cola Zero^®^, Rivella blue^®^); - Sweeteners in tablet/powder form (e.g. saccharin, stevia); - Protein shakes (as a meal replacement or sports nutrition); - Chips/crisps or aperitif biscuits (**9**). | - Artificial sweeteners (Assugrin^®^, aspartame); - Bran (**2**). |

^a^ Food groups are classified according to the stages of the Swiss food-based dietary guidelines (Swiss Food Pyramid): <https://www.blv.admin.ch/blv/en/home/lebensmittel-und-ernaehrung/ernaehrung/empfehlungen-informationen/schweizer-ernaehrungsempfehlungen.html>

**Additional file 2: Comparison between group-level differences (bias, %) and Spearman’s correlation coefficients in nutrient intakes estimated by the eFFQ and the paper-based FFQ among all participants (n=75, same results as Table 2) vs. participants with confirmed order of FFQ completion (n=66).**

|  | **All participants (n=75)** | | |  | **Participants with confirmed order of FFQ completion (n=66)** | | |
| --- | --- | --- | --- | --- | --- | --- | --- |
| **Daily intake** | % Group-level  differences (medians) ^a^ | % Group-level  differences (means) ^b^ | SCC |  | % Group-level  differences (medians) ^a^ | % Group-level  differences (means) ^b^ | SCC |
| Energy (kcal) | 5.3 | -0.9 | 0.67 |  | 6.6 | -1.2 | 0.67 |
| Protein (E%) | 4.2* | 5.0 | 0.61 |  | 5.5* | 5.5 | 0.60 |
| Carbohydrates (E%) | -6.3* | -6.3 | 0.69 |  | -6.2* | -6.3 | 0.69 |
| Total sugars (disaccharides) (g) | 9.1 | -1.0 | 0.75 |  | 9.6 | -2.4 | 0.75 |
| Dietary fibre (g) | 37.2* | 28.6 | 0.64 |  | 39.2* | 28.9 | 0.61 |
| Total fat (E%) | 0.0 | 0.3 | 0.54 |  | -0.2 | -0.2 | 0.55 |
| Saturated fatty acids (E%) | 2.7 | -0.5 | 0.45 |  | 3.1 | 0.0 | 0.46 |
| Monounsaturated fatty acids (E%) | -1.9* | -6.6 | 0.58 |  | -4.0* | -7.7 | 0.60 |
| Polyunsaturated fatty acids (E%) | 27.5* | 22.5 | 0.48 |  | 27.0* | 22.0 | 0.48 |
| Cholesterol (mg) | -25.7* | -26.7 | 0.74 |  | -24.8* | -26.4 | 0.76 |
| Alcohol (g) | 119.0* | 50.0 | 0.86 |  | 120.4* | 52.7 | 0.88 |
| Vitamin A (µg-RE ^c^) | -1.0* | 4.4 | 0.59 |  | -4.6 | 3.4 | 0.55 |
| Vitamin D (calciferol, µg) | -0.1 | -3.3 | 0.56 |  | -1.1 | -5.4 | 0.56 |
| Calcium (mg) | -13.3* | -21.9 | 0.54 |  | -13.0* | -20.7 | 0.52 |
| Iron (mg) | -11.0* | -14.1 | 0.57 |  | -8.5* | -13.8 | 0.58 |

FFQ: Food frequency questionnaire; SCC, Spearman’s rank correlation coefficients (p<0.001 for all nutrients and both populations); E%, percentage of total energy intake; RE, retinol equivalent.

^a^ Group-level differences were based on medians [(median intake eFFQ) / (median intake paper-based FFQ) * 100 - 100)]. Wilcoxon signed-rank tests were used to assess differences between the two FFQs (*P<0.05).

^b^ Group-level differences were based on means [(mean intake eFFQ) / (mean intake paper-based FFQ) * 100 - 100)].

^c^ 1 μg RE equals 1 μg of retinol + 12 μg of β-carotene for the paper-based FFQ and 1 μg of retinol + 6 μg of β-carotene + 12 μg of other provitamin A carotenoids for the eFFQ.

**Additional file 3: Scatter plots of the energy and three macronutrient intakes estimated by the new eFFQ and the old paper-based FFQ (n=75).**

Solid blue diagonal lines represent the identity function y=x and r = Spearman’s correlation coefficients (all P-values < 0.001). FFQ, Food Frequency Questionnaire.
